# Aspartate Metabolism Facilitates IL-1β Production in Inflammatory Macrophages

**DOI:** 10.3389/fimmu.2021.753092

**Published:** 2021-10-21

**Authors:** Hao Wang, Xueyue Zheng, Bingnan Liu, Yaoyao Xia, Zhongquan Xin, Baichuan Deng, Liuqin He, Jinping Deng, Wenkai Ren

**Affiliations:** ^1^State Key Laboratory for Conservation and Utilization of Subtropical Agro-Bioresources, Guangdong Laboratory of Lingnan Modern Agriculture, Guangdong Provincial Key Laboratory of Animal Nutrition Control, National Engineering Research Center for Breeding Swine Industry, College of Animal Science, South China Agricultural University, Guangzhou, China; ^2^Hunan Provincial Key Laboratory of Animal Intestinal Function and Regulation, Laboratory of Animal Nutrition and Human Health, College of Life Sciences, Hunan Normal University, Changsha, China; ^3^Institute of Subtropical Agriculture, Chinese Academy of Sciences, Changsha, China

**Keywords:** asparagine, aspartate, HIF-1α, inflammasome, macrophage

## Abstract

Increasing evidence support that cellular amino acid metabolism shapes the fate of immune cells; however, whether aspartate metabolism dictates macrophage function is still enigmatic. Here, we found that the metabolites in aspartate metabolism are depleted in lipopolysaccharide (LPS) plus interferon gamma (IFN-γ)-stimulated macrophages. Aspartate promotes interleukin-1β (IL-1β) secretion in M1 macrophages. Mechanistically, aspartate boosts the activation of hypoxia-inducible factor-1α (HIF-1α) and inflammasome and increases the levels of metabolites in aspartate metabolism, such as asparagine. Interestingly, asparagine also accelerates the activation of cellular signaling pathways and promotes the production of inflammatory cytokines from macrophages. Moreover, aspartate supplementation augments the macrophage-mediated inflammatory responses in mice and piglets. These results uncover a previously uncharacterized role for aspartate metabolism in directing M1 macrophage polarization.

## Introduction

Immune cells are central determinants of the maintenance of physiological homeostasis by protecting the host from environmental stimuli (1). Once immune cells sense pathogeny or other insults, they activate and proliferate rapidly to perform the corresponding immune functions (2). Multiple contributors are associated with the fate of immune cells. Notably, by providing energy and substrates, metabolic pathways and metabolites are vital in modulating immune cell growth and survival and in instructing the effector function of immune cells (2). For example, altered amino acid metabolism (e.g., arginine metabolism) is one of characteristics employed to define macrophage polarization (3–7).

Essential amino acids, which cannot be sufficiently synthesized by the body and must obtained from dietary sources, are crucial for the function of immune cells (8). For instance, methionine is critical for T-cell activation (9), and serine or tryptophan metabolism is associated with macrophage biology (10, 11). Non-essential amino acids can be synthesized endogenously, but they switch into being conditionally essential when the synthesis rates cannot meet the demand of the host (8, 12). Notably, the activation of immune cells triggers drastic changes in transcription and translation to produce effector molecules, which must reprogram their metabolism to support these huge metabolic demands (13). In this case, non-essential amino acids (NEAAs) may become conditionally essential for the activation of immune cells. Previous reports have demonstrated that glutamate and cysteine regulate the proliferation and/or activation of T cells (14, 15), macrophages (16), B cells (17), and dendritic cells (DCs) (18). Moreover, we have also illuminated the roles of glutamine metabolism in T helper 17(Th 17) cells, regulatory T cells (Tregs) (19), and macrophages (15). As an important NEAA for nucleotide synthesis (20), aspartate (Asp) has critical roles in cancer and proliferative immune cells (e.g., T cells) (21–24); however, little is known about its physiological function in non-proliferating immune cells (chiefly macrophages). More importantly, no prior studies have investigated the metabolic cues and key regulatory function of Asp metabolism in directing macrophage polarization.

Here, we found an unexpected role of Asp metabolism in promoting interleukin-1β (IL-1β) production from inflammatory macrophages by rewiring cellular metabolism and boosting the activation of hypoxia-inducible factor-1α (HIF-1α) and inflammasome signaling. We propose that Asp metabolism is important to fine-tune macrophage-mediated inflammation.

## Materials and Methods

### Animals and Antibodies

All experiments were approved by the Administration Office of Laboratory Animals of South China Agricultural University. ICR female mice (6–8 weeks old) were housed with free food and water in a room with standard day/night cycle conditions. Piglets were purchased from Hunan New Wellful Co., Ltd. (Changsha, China). Antibodies against IL-1β (12426s), mammalian target of rapamycin (mTOR; 2972s), p-mTOR (5536s), and HIF-1α (14179s) were purchased from Cell Signaling Technology (Danvers, MA, USA). Antibodies against IκB kinase (IKK; Sc-7607), p-IKK (sc-293135), p-IκB (Sc-8404), and apoptosis-associated speck like protein containing a caspase recruitment domin (ASC; Sc-514414) were purchased from Santa Cruz Biotechnology, Inc. (Dallas, TX, USA). Antibodies against IκB (51066-1-AP), p65 (10745-1-AP), and β-actin (60008-1-Ig) were purchased from Proteintech (Rosemont, IL, USA). The antibody against p-p65 (bs-0982R) was purchased from Bioss (Beijing, China). Antibodies against Nod-like receptor protein 3 (NLRP3; ab214185) and caspase-1 (ab179515) were purchased from Abcam (Cambridge, UK).

### Peritoneal Macrophage Isolation and Culture

Peritoneal macrophages were isolated as in our previous study (10). Mice were injected intraperitoneally with 2 ml of 4% sterile thioglycolate, and peritoneal macrophages were collected *via* peritoneal lavage with 5 ml cold phosphate-buffered saline (PBS). Peritoneal lavage was spun down for 5 min at 1,200 rpm and resuspended in Dulbecco’s modified Eagle’s medium (DMEM) containing 10% fetal bovine serum (FBS) and 1% penicillin–streptomycin. Non-adherent cells were removed 3 h later through washing twice with sterile PBS, whereas adherent cells were cultured in complete DMEM.

### Treatments in Macrophages

Peritoneal macrophages were stimulated with 1 μg/ml lipopolysaccharide (LPS) (L2630; Sigma, St. Louis, MO, USA) plus 20 ng/ml interferon-γ (IFN-γ) (315-05; PeproTech, Cranbury, NJ, USA) for 15 h to induce M1 polarization. In some experiments, Asp, asparagine, cytosine, or deoxycytidine was added to DMEM in the indicated dosages (0.1, 1, or 10 mM for Asp or asparagine and 100 μM for cytosine or deoxycytidine). PX-478, MCC-950, VX-765, and IKK16 were purchased from Selleck Chemicals (Houston, TX, USA). Dactinomycin and cycloheximide were purchased from MedChemExpress (Monmouth Junction, NJ, USA). The cells were treated with PX-478 (10 μM), MCC-950 (10 μM), VX-765 (20 μM), dactinomycin (2 μg/ml), or cycloheximide (10 μM) for 15 h when treated with LPS and IFN-γ in order to inhibit HIF-1α, NLRP3, and caspase-1 transcription or translation, respectively. The cells were treated with the IKK inhibitor IKK-16 (10 μM) for 20 min before treated with LPS and IFN-γ.

### *Citrobacter rodentium* Infection of Mice

Six weeks old C57 female mice were infected by oral gavage with 200 μl of PBS containing approximately 1 × 10^8^ colony forming units (CFUs) of *Citrobacter rodentium* (DBS100). The colons of all mice were collected for further analysis at 10 days post-infection. The colons were homogenized in PBS and then serial diluted and inoculated on selective MacConkey agar. Bacteria were enumerated after growth overnight at 37°C.

### Aspartate Supplementation in Piglets

A total of 44 six-week-old piglets were assigned into four groups: Con group (basal diet), 0.005% Asp group (basal diet with 0.005% Asp supplementation), 0.05% Asp group (basal diet with 0.05% Asp supplementation), and 0.5% Asp group (basal diet with 0.5% Asp supplementation). The piglets were kept individually in an environment-controlled facility with hard plastic slatted flooring and with free access to food and drinking water. Five piglets per group were sacrificed on day 70 and six piglets per group sacrificed on day 142. The tissues from the jejunum, ileum, and colon of piglets were collected immediately and stored at −80°C.

### Bactericidal Activity Assay

For the bactericidal activity assays, 1 × 10^6^ cells/well macrophages were incubated with 1 × 10^7^ ETEC (*Escherichia coli* strain SEC470) for 3 h on a six-well chamber slide. After infection, the macrophages were washed with cold PBS to stop additional bacterial uptake, and the extracellular bacteria were completely killed with 300 μg/ml gentamicin for 30 min. After the clearance of gentamicin with PBS, 500 μl 1% Triton X-100 was used for cell lysis, and 100 μl diluted lysate was inoculated on Luria–Bertani (LB) agar plates and incubated at 37°C in a CO_2_ incubator overnight to count and calculate the number of CFUs.

### RNA Extraction and Quantitative Real-Time PCR

RNA was isolated from peritoneal macrophages using the EZ-press RNA Purification Kit (B0004D) purchased from EZBioscience^®^ (Roseville, MN, USA). RNA was quantified and 500 ng of RNA was reverse transcribed using the Color Reverse Transcription Kit (A0010CGQ; EZBioscience^®^). Real-time PCR was performed using 2× Color SYBR Green qPCR Master Mix (A0012-R2, EZBioscience^®^) on the QuantStudio 6 Real-Time PCR System (Thermo Fisher Scientific, Waltham, MA, USA). Fold change was assessed using the 2^−ΔΔ^*^C^*^t^ method with β-actin. The primers used in this study are shown in **Supplementary Table S1**.

### Western Blot

Western blot was performed according to our previous studies (10, 25). The cells were lysed in ice-cold RIPA for 10 min. The bicinchoninic acid (BCA) protein assay kit (Beyotime Biotechnology, Shanghai, China) was used to quantify the protein concentration. Then, equal amounts of protein were subjected to 10% sodium dodecyl sulfate polyacrylamide gel electrophoresis (SDS-PAGE). The proteins were transferred onto polyvinylidene difluoride (PVDF) membranes (Bio-Rad, Hercules, CA, USA), and blocked with 5% bovine serum albumin (BSA) Tris/Tween-buffered saline buffer (TBST) for 1 h. Then, the proteins were incubated with primary and secondary antibodies and visualized using a chemiluminescent reagent. The signal intensity was digitally quantified and normalized to actin protein abundance.

### Cytokine Measurement

IL-1β and tumor necrosis factor alpha (TNF-α) on supernatants obtained from cell cultures were quantified by ELISA (#KE10003 and #KE10002; Proteintech) based on protocol from the manufacturer.

### Metabolite Profiling Analysis

The intracellular and supernatant levels of the metabolites of macrophages were quantified through liquid chromatography–mass spectrometry (LC-MS), as in our previous study (25). Approximately 1 × 10^7^ cells/well macrophages were plated in six-well plates, treated as indicated, and collected for analysis. Briefly, the cells were washed with cold PBS and collected with a cell scraper (Thermo Fisher Scientific). Then, 1 ml extract solution (50% methanol acetonitrile) was added into each sample. The samples were homogenized for 2 min, sonicated at 35 Hz in ice water for 10 min, and centrifuged at 14,500 rpm for 15 min at 4°C. The supernatant was dried in a vacuum concentrator at 50°C, and re-dissolved with 200 μl 50% methanol water. Subsequently, the samples were homogenized for 2 min, sonicated at 35 Hz in ice water for 10 min, and centrifuged at 14,500 rpm for 15 min at 4°C. Then, the supernatant was transferred into a fresh glass vial for LC-MS analysis.

### Statistical Analysis

All data were analyzed and prepared with GraphPad Prism v.8.0. All data displayed in graphs are expressed as the mean ± SEM. A *t*-test was used to analyze the data with a Gaussian distribution and had equal variance. Unpaired *t*-test with Welch’s correction was used to analyze the data with a Gaussian distribution but without equal variance. A non-parametric test (Mann–Whitney *U* test) was used to analyze the data that were not normally distributed. One-way ANOVA and Dunnett’s multiple comparisons were used to analyze the data that have more than two groups, with a Gaussian distribution, and had equal variance. Kruskal–Wallis and Dunn’s multiple comparisons tests were used to analyze the data that were not normally distributed. The D’Agostino–Pearson omnibus normality test and the Kolmogorov–Smirnov test were used to analyze the Gaussian distribution of the data. The homogeneity of variance test or the Brown–Forsythe test was used to analyze the variance of data. A significant difference was defined as *p* < 0.05.

## Results

### Aspartate and Its Metabolites Are Depleted in M1 Macrophages

**Figure 1:**
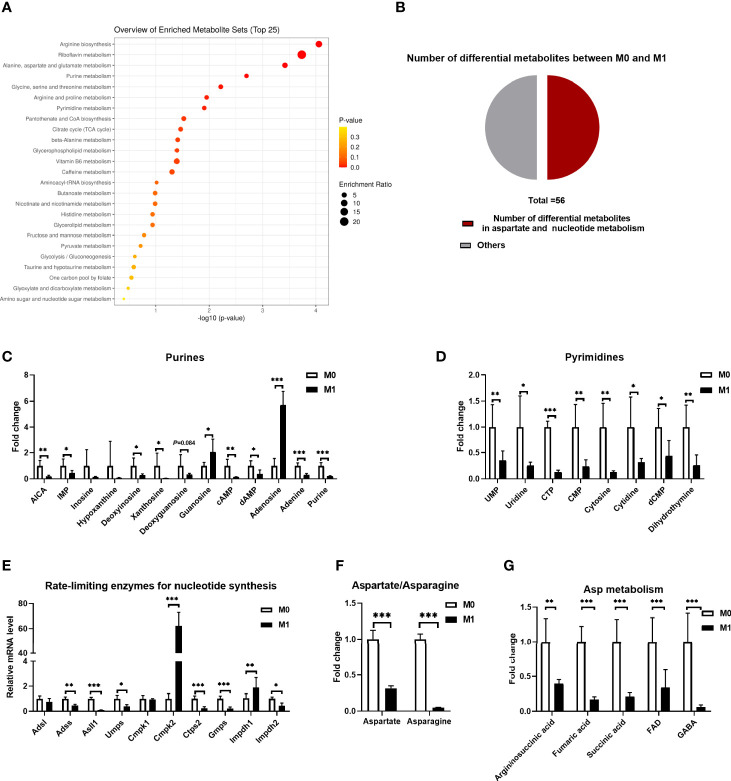
M1 macrophages show altered aspartate metabolism. **(A)** Kyoto Encyclopedia of Genes and Genomes (KEGG) annotation and enrichment of differential metabolites between unstimulated (M0) and lipopolysaccharide (LPS)/interferon gamma (IFN-γ)-stimulated (M1) macrophages (*n* = 6). **(B)** Pie chart of the different metabolites between M0 and M1 macrophages (*n* = 6). **(C, D)** Fold change of intracellular purine **(C)** and pyrimidine **(D)** between M0 and M1 macrophages (*n* = 6). **(E)** mRNA expressions of rate-limiting enzymes for nucleoside synthesis between M0 and M1 macrophages (*n* = 6). **(F)** Fold change of intracellular aspartate and asparagine between M0 and M1 macrophages (*n* = 6). **(G)** Fold change of intracellular metabolites for aspartate synthesis between M0 and M1 macrophages (*n* = 6). Data in **(A–D)** and **(F, G)** were adapted from **Figure 3A** of our previous study (27). All data were analyzed with unpaired *t*-test. *Error bars* represent the mean ± SEM. *p ≤ 0.05, **p ≤ 0.01, ***p ≤ 0.001.

Macrophages reprogram their metabolism to instruct their effector functions, such as producing mediators and cytokines (26). With LC-MS, our previous study showed a remarkable difference in the cellular metabolism between resting macrophages (M0) and macrophages stimulated with LPS plus IFN-γ (27). We reanalyzed the differential metabolites and found that most of them were enriched in glycolysis, tricarboxylic acid (TCA) cycle, and amino acid metabolism (**Figure 1A**). Notably, half of them could be incorporated into Asp and nucleotide metabolism (**Figure 1B**), including Asp, asparagine, purines, and pyrimidines (**Figures 1C, D, F, G**). Particularly, the levels of the majority of these metabolites in M1 macrophages were decreased compared to those in M0 macrophages, except for guanosine and adenosine (**Figures 1C, D**). Besides, the gene expressions of the rate-limiting enzymes in purine and pyrimidine synthesis were also decreased, in addition to *Cmpk2* and *Impdh1* (**Figure 1E**). Also, the messenger RNA (mRNA) expressions of the genes related to Asp synthesis and transportation were changed (**Supplementary Figure S1**). Collectively, M1 macrophage polarization shows remarkable change in cellular metabolism, especially Asp metabolism.

### Aspartate Promotes IL-1β Secretion From M1 Macrophages

**Figure 2:**
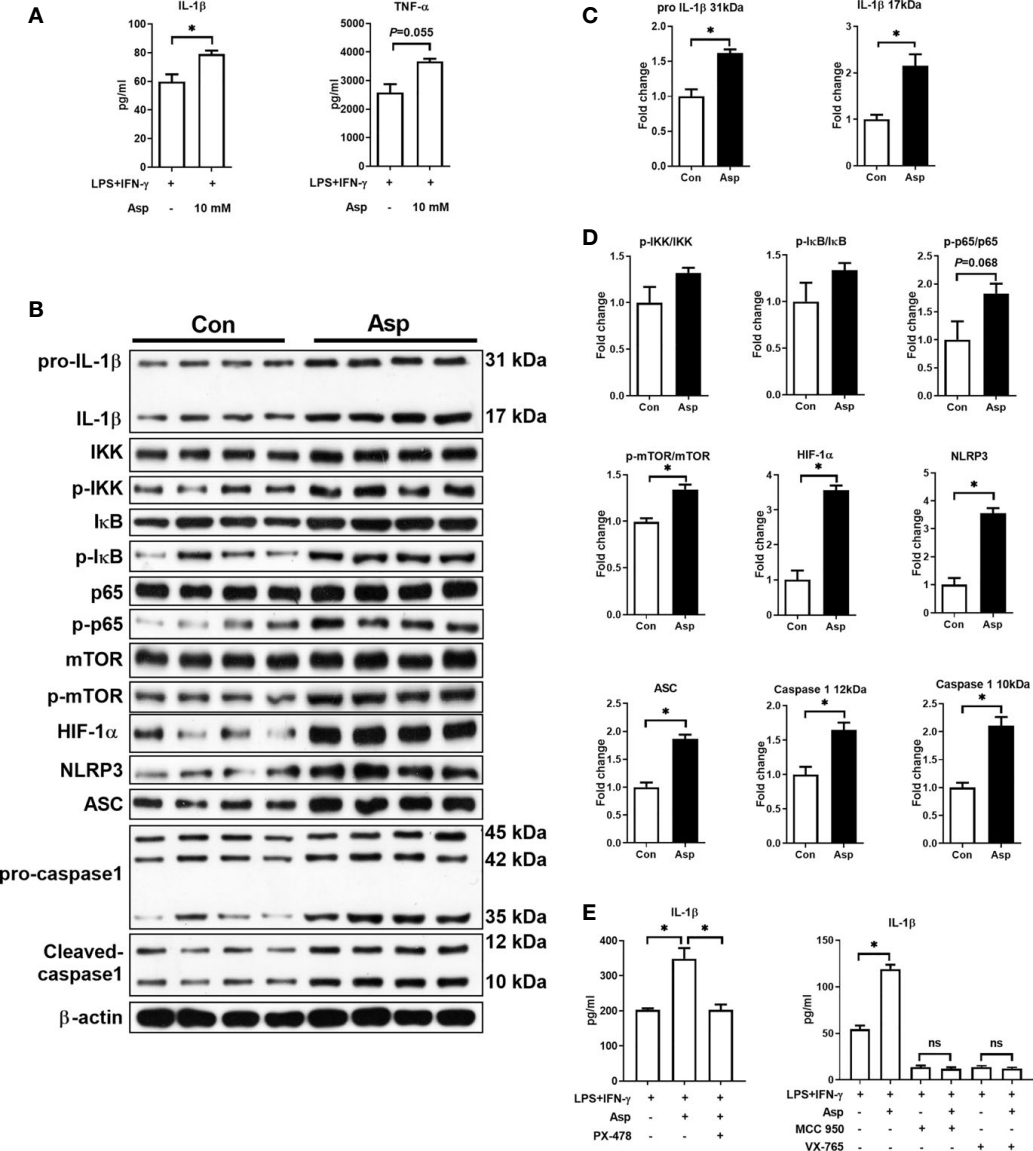
Aspartate promotes the production of interleukin 1β (IL-1β) and the activation of signal pathways in macrophages. **(A)** IL-1β and tumor necrosis factor alpha (TNF-α) secretion in macrophages (*n* = 5). **(B–D)** Protein abundance of IL-1β, IκB kinase (IKK), p-IKK, IκB, p-IκB, p65, p-65, mammalian target of rapamycin (mTOR), p-mTOR, S6K1, p-S6K1, hypoxia-inducible factor-1α (HIF-1α), Nod-like receptor protein 3 (NLRP3), apoptosis-associated speck like protein containing a caspase recruitment domin (ASC), and caspase-1 in macrophages (*n* = 4). **(E)** IL-1β secretion in M1 macrophages (*n* = 3–4). Data were analyzed with unpaired *t*-test **(A–D)** or with one-way ANOVA **(E)**. *Error bars* represent the mean ± SEM. ns, not significant. *p ≤ 0.05.

Asp is a key substrate in nucleotide and protein synthesis, but cellular Asp availability is largely dependent on mitochondrial respiration (24). However, M1 macrophages are characterized as a dysfunctional electron transport chain (28), which might result in insufficient Asp metabolism and inhibit the synthesis of pro-inflammatory cytokines. Thus, we then explored the influence of Asp on the polarization of M1 macrophages. Our results showed that exogenous Asp supplementation promoted the secretion of IL-1β and TNF-α (**Figure 2A** and **Supplementary Figure S2A**). However, Asp had little effect on the mRNA expressions of IL-1β and TNF-α (**Supplementary Figure S2B**). As IL-1β has been shown to be directly involved in the production of multiple pro-inflammatory mediators (29), and is specifically secreted by inflammatory macrophages (30), we thus focused on the role of Asp in the IL-1β production of M1 macrophages in the subsequent study.

Nuclear factor kappa-B (NF-κB) and the NLRP3 inflammasome are two key pathways involved in M1 polarization (31, 32). Moreover, mTOR and HIF-1α signaling are also associated with macrophage polarization (10, 33, 34). Thus, we analyzed the activation of these pathways. Aspartate increased the protein levels of pro-IL-1β (31 kDa) and IL-1β (17 kDa) (**Figures 2B, C**). As expected, Asp significantly promoted the activation of mTOR, HIF-1α, and NLRP3 inflammasome (including NLRP3, ASC, and caspase-1), while it had little effect on the NF-κB pathway (**Figures 2B, D**). These results indicated that Asp promotes M1 polarization through the activation of these pathways. To validate this hypothesis, PX-478, MCC-950, and VX-765 were used to inhibit HIF-1α, NLRP3, and caspase-1 in Asp-treated M1 macrophages, respectively. These inhibitors significantly blocked the effect of Asp on IL-1β secretion (**Figure 2E**). Collectively, Asp-enhanced M1 macrophage polarization is largely dependent on the activation of HIF-1α and NLRP3 inflammasome.

### Aspartate Rewires Cellular Metabolism of M1 Macrophages

**Figure 3:**
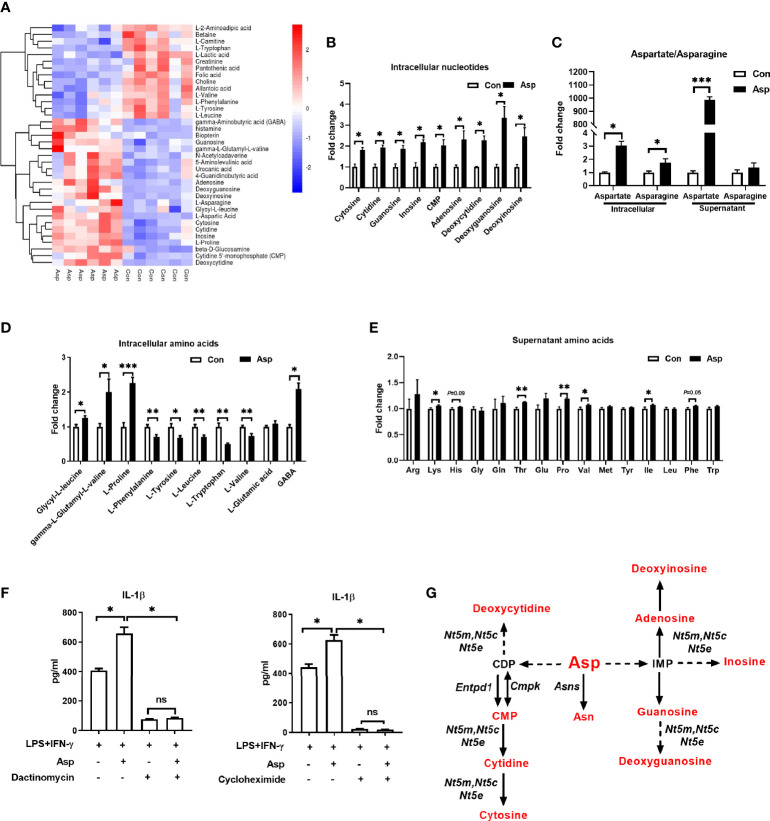
Aspartate remodels the metabolism of macrophages. **(A)** Heatmap analysis of the different metabolites between M1 macrophages and aspartate-treated M1 macrophages (*n* = 6). **(B)** Fold change of intracellular nucleotide in M1 macrophages and aspartate-treated M1 macrophages (*n* = 6). **(C)** Fold change of the supernatant and intracellular asparagine/aspartate in M1 macrophages and aspartate-treated M1 macrophages (*n* = 6). **(D)** Fold change of intracellular amino acids in M1 macrophages and aspartate-treated M1 macrophages (*n* = 6). **(E)** Fold change of supernatant amino acids in M1 macrophages and aspartate-treated M1 macrophages (*n* = 6). **(F)** Secretion of interleukin 1β (IL-1β) from M1 macrophages with aspartate supplementation combined with dactinomycin or cycloheximide (*n* = 3–4). **(G)** Different metabolites in aspartate metabolism between M1 macrophages and aspartate-treated M1 macrophages (*n* = 6). Data were analyzed with unpaired *t*-test **(B–E)** or with one-way ANOVA **(F)**. *Error bar*s represent the mean ± SEM. ns, not significant. *p ≤ 0.05, **p ≤ 0.01, ***p ≤ 0.001.

Given that the metabolic output is closely associated with macrophage polarization (35), M1 macrophage polarization showed remarkable change in Asp metabolism (**Figure 1**). Thus, we asked whether Asp facilitates M1 macrophage polarization through altering intracellular metabolism. Here, we performed metabolomics to assay the intracellular metabolites in macrophages cultured with Asp. The cellular metabolites of M1 macrophages cultured with Asp-supplemented media were obviously different from those in M1 macrophages cultured with normal medium (**Figure 3A** and **Supplementary Figures S2C, D**). Similarly, the differential metabolites were also enriched in Asp metabolism, including Asp, asparagine, and nucleotides (**Figures 3B, C**). However, Asp had little effect on the mRNA expressions of the enzymes for nucleotide metabolism (**Supplementary Figure S2E**). Intriguingly, Asp significantly affected the amino acid metabolism and the aminoacyl-tRNA biosynthesis (**Figures 3D, E** and **Supplementary Figure S2F**). Aminoacyl-tRNA biosynthesis is an essential biological process responsible for charging amino acids to their cognate transfer RNAs (tRNAs) in order to provide the substrates for global protein synthesis (36), suggesting that Asp promotes the secretion of IL-1β through translation. Thus, we added dactinomycin and cycloheximide to inhibit transcription and translation, respectively. Both dactinomycin and cycloheximide were sufficient to block the Asp-mediated IL-1β secretion (**Figure 3F**). Collectively, these results suggest that Asp promotes the production of IL-1β through metabolic remodeling.

### Asparagine Accelerates IL-1β Secretion From M1 Macrophages

Aspartate is the key precursor for the synthesis of nucleotides and asparagine (22). Moreover, this study revealed that Asp metabolites were decreased in M1 macrophages (**Figure 1B**), while supplementation of Asp reversed this (**Figure 3G**). Therefore, we explored whether Asp regulates macrophage polarization through its derivatives. Strikingly, the targeted LC-MS analysis showed that intracellular nucleoside was decreased (**Figure 1D**), but the supernatant cytosine and deoxycytidine were increased in M1 macrophages (**Supplementary Figure S3A**). In addition, Asp also increased the supernatant cytosine and deoxycytidine (**Supplementary Figure S3B**). Then, we determined whether cytosine and deoxycytidine could contribute to the polarization of M1 macrophages. However, cytosine or deoxycytidine was not able to increase the abundance of IL-1β and caspase-1 and the phosphorylation of IKK (**Supplementary Figures S3C, D**).

**Figure 4:**
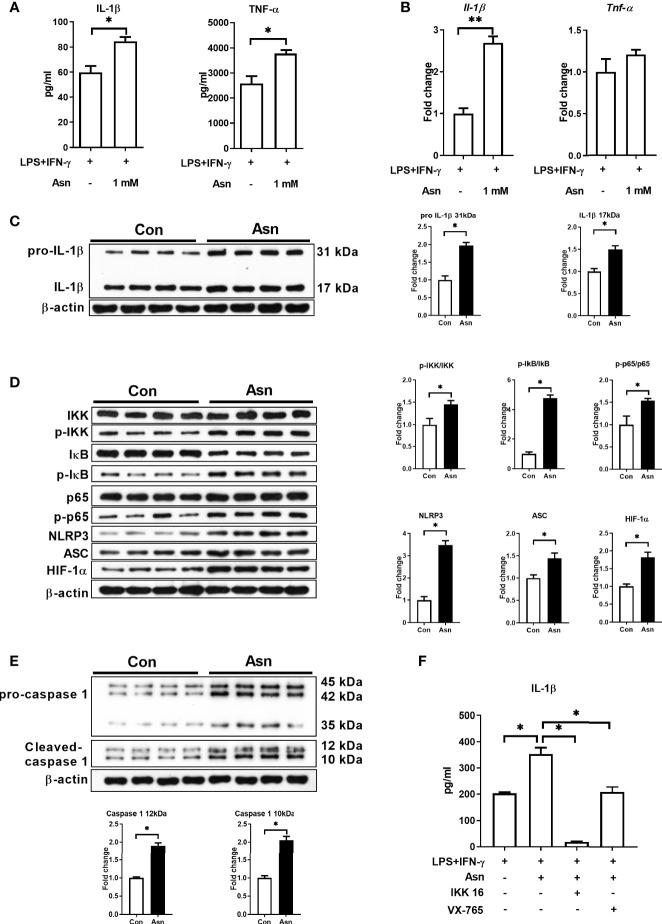
Asparagine promotes the production of interleukin 1β (IL-1β) and the activation of signal pathways in macrophages. **(A)** IL-1β and tumor necrosis factor alpha (TNF-α) secretion in M1 macrophages and asparagine-treated M1 macrophages (*n* = 4–5). **(B)** mRNA expressions of IL-1β and TNF-α in M1 macrophages and asparagine-treated M1 macrophages (*n* = 6). **(C–E)** Protein abundance of IL-1β, IκB kinase (IKK), p-IKK, IκB, p-IκB, p65, p-65, hypoxia-inducible factor-1α (HIF-1α), Nod-like receptor protein 3 (NLRP3), apoptosis-associated speck like protein containing a caspase recruitment domin (ASC), and caspase-1 in macrophages (*n* = 4). **(F)** IL-1β secretion in M1 macrophages with asparagine combined with IKK16 or VX-765 (*n* = 3). Data were analyzed with unpaired *t*-test **(A–E)** or with one-way ANOVA **(F)**. *Error bars* represent the mean ± SEM. *p ≤ 0.05, **p ≤ 0.01.

We next explored the role of asparagine in M1 macrophages. Our results showed that 1 mM asparagine was sufficient to promote the production of IL-1β and TNF-α in M1 macrophages (**Figures 4A–C** and **Supplementary Figures S4A, B**). To determine whether asparagine promotes the production of IL-1β in M1 macrophages by the same mechanism as Asp, we examined the activation of the associated signaling pathways. Interestingly, asparagine promoted the activation of NF-κB, HIF-1α, and NLRP3 inflammasome (**Figures 4D, E**). Furthermore, IKK16 (an inhibitor of IκB) and VX 765 (an inhibitor of HIF-1α) significantly reduced the production of IL-1β in M1 macrophages cultured with asparagine (**Figure 4F**). Taken together, these data suggest that Asp promotes M1 macrophage polarization possibly through asparagine.

### Asparagine Rewires Cellular Metabolism to Support IL-1β Production

**Figure 5:**
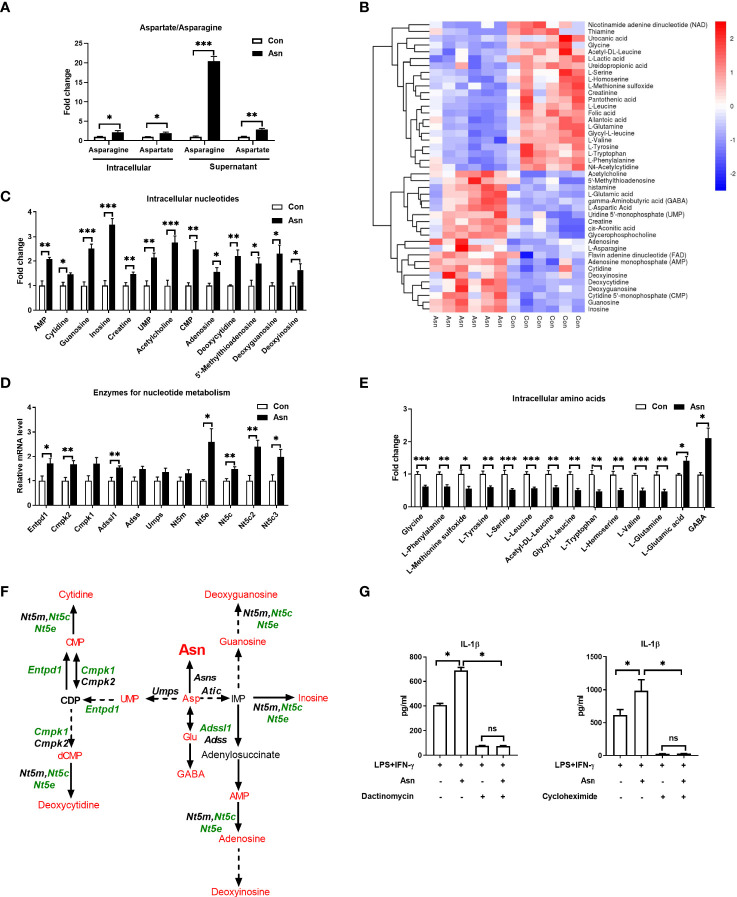
Asparagine modulates the cellular metabolism of macrophages. **(A)** Fold change of intracellular and supernatant asparagine/aspartate in M1 macrophages and asparagine-treated M1 macrophages (*n* = 6). **(B)** Heatmap analysis of the different metabolites between M1 macrophages and asparagine-treated M1 macrophages (*n* = 6). **(C)** Fold change of the intracellular nucleotides in M1 macrophages and asparagine-treated M1 macrophages (*n* = 6). **(D)** mRNA expressions of the rate-limiting enzymes for nucleoside synthesis in M1 macrophages and asparagine-treated M1 macrophages (*n* = 6). **(E)** Fold change of the intracellular amino acids in M1 macrophages and asparagine-treated M1 macrophages (*n* = 6). **(F)** Different metabolites in aspartate metabolism between M1 macrophages and asparagine-treated macrophages (*n* = 6). **(G)** Secretion of interleukin 1β (IL-1β) from M1 macrophages with asparagine supplementation combined with dactinomycin or cycloheximide (*n* = 4). Data were analyzed with unpaired *t*-test **(A, C–E)** or with one-way ANOVA **(G)**. *Error bars* represent the mean ± SEM. ns, not significant. *p ≤ 0.05, **p ≤ 0.01, ***p ≤ 0.001.

Considering that Asp is the precursor of asparagine, and asparagine supplementation increased the supernatant and intracellular levels of Asp and asparagine (**Figure 5A**), we investigated whether asparagine also affects the polarization of macrophages through intracellular metabolic remodeling. Similar to the effects of Asp, asparagine modulated the nucleotide and amino acid metabolism in M1 macrophages and induced the activation of aminoacyl-tRNA biosynthesis (**Figures 5B–F** and **Supplementary Figures S5A–D**). Also, the inhibition of transcription or translation significantly blocked the effect of asparagine on the production of IL-1β in macrophages (**Figure 5G**). These data support the hypothesis that asparagine modulates nucleotide and amino acid metabolism to support M1 macrophage polarization.

### Aspartate Augments Macrophage-Mediated Inflammatory Responses *In Vivo*

**Figure 6:**
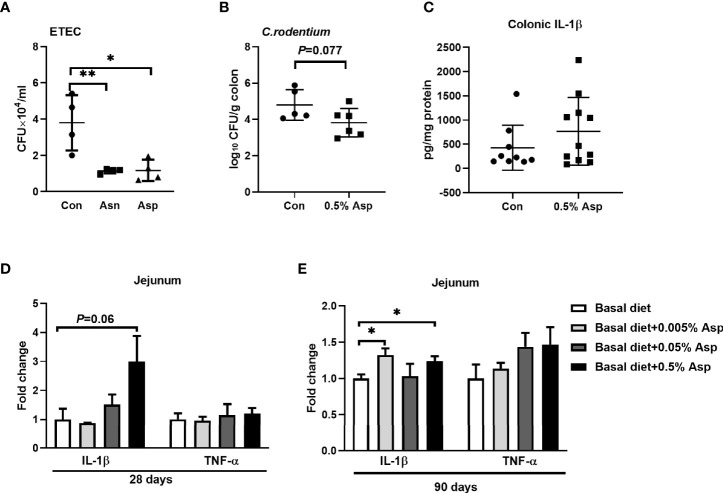
Aspartate affects the immune responses in mice and piglets. **(A)** Phagocytosis in macrophages (*n* = 3–4). **(B)** The *Citrobacter rodentium* count in the colon from control mice and mice with 0.5% aspartate supplementation (*n* = 5–6). **(C)** Interleukin 1β (IL-1β) levels in the colon from control mice and mice with 0.5% aspartate supplementation (*n* = 8–11). **(D)** mRNA expressions of IL-1β and tumor necrosis factor alpha (TNF-α) in the jejunum from control piglets and piglets given aspartate for 28 days (*n* = 4–5). **(E)** mRNA expressions of IL-1β and TNF-α in the jejunum from control piglets and piglets with aspartate supplementation for 90 days (*n* = 5–6). Data were analyzed with unpaired *t*-test **(A–C)** or with one-way ANOVA **(D, E)**. *Error bars* represent the mean ± SEM. *p ≤ 0.05, **p ≤ 0.01.

To identify whether Asp and asparagine affect the bactericidal activity of macrophages, we assessed their effects on the bactericidal activity of macrophages against ETEC. The number of engulfed bacteria in the macrophages was determined at 3 h post-infection. Aspartate or asparagine enhanced the bactericidal activity of macrophages (**Figure 6A**). Moreover, *C. rodentium* has been shown to induce the activation of the NLRP3 inflammasome in macrophages, and IL-1β plays a critical role in the defense against *C. rodentium* infection (37, 38). To identify the role of Asp in the clearance of *C. rodentium*, we gave mice drinking water supplemented with Asp at a dosage of 0.5 mg/ml for 2 weeks before they were orally infected with 10^8^ CFU/mouse. Although the body weight and the length of the colon were similar between the two groups (**Supplementary Figures S6A, B**), a lower bacterial burden was observed in the colon of mice receiving Asp (**Figure 6B**). Aspartate increased the baseline level of IL-1β in the colon, but no significant difference was observed between the two groups (**Figure 6C**). The possible explanation is that the colonic IL-1β analyzed is derived from a variety of immune cells [e.g., neutrophils (39) and macrophages (10)]; the effects of Asp on these cells are inconsistent. These results indicate that Asp promotes the bactericidal activity of macrophages in the context of bacterial infection.

Infants are susceptible to pathogen infection due to the immature development of their gastrointestinal tract and the immature function of their underdeveloped immune system, leading to diarrhea and even death (40). Thus, it is important to maintain the intestinal immune balance of a newborn host. Considering that macrophage polarization plays an important role in maintaining intestinal immune balance (41), we wanted to uncover whether Asp could alter the intestinal immune status of a newborn host. The piglet is similar to a human in terms of anatomy and physiology, and it is a more excellent animal model for experiments than rodents (42). Therefore, weaned piglets were used as the models to investigate the role of Asp in intestinal immune responses. As expected, diet supplementation with Asp enhanced the intestinal immune responses, especially the IL-1β expression in the jejunum, in a dosage- and time-dependent manner (**Figures 6D, E** and **Supplementary Figures S6C, D**). Taken together, Asp confers protection against intestinal bacterial infections by enhancing the macrophage-mediated inflammatory responses *in vivo*.

## Discussion

The metabolic remodeling of immune cells (e.g., amino acid metabolism) (4) dramatically influences host homeostasis and immunity (6). Amino acids [e.g., arginine (3) and glutamine (15, 19)] can be used as energy metabolic substrates and signal molecules and can fuel other metabolic pathways to regulate the survival and function of immune cells (5). Increasing pieces of evidence have shown that Asp metabolism is involved in the survival of cancer cells (22) and the activation of proliferative immune cells (e.g., T cells) (20, 43, 44), but the role of Asp in non-proliferating cells is not fully understood. This study found that Asp promoted M1 macrophage polarization through metabolic remodeling and the activation of HIF-1α and NLRP3 inflammasome components in peritoneal macrophages. The Asp derivative, asparagine, but not nucleotides, also remodeled the cellular metabolism and activated HIF-1α and inflammasome signaling to boost IL-1β secretion from M1 macrophages.

Macrophages are known to display remarkable heterogeneity and plasticity (45). The polarization of macrophages into an inflammatory or an anti-inflammatory phenotype showed distinct metabolic requirements (e.g., amino acids) (46). Blood Asp levels were low and the transport of Asp into most mammalian cells was inefficient (28, 47); thus, the availability of cellular Asp and Asp-derived metabolites are largely dependent on the TCA cycle flux and mitochondrial respiration (24). However, the M1 phenotype showed a broken TCA cycle and a dysfunctional electron transport chain (48). Consistent with these results, we showed that the levels of Asp and Asp-derived metabolites, including asparagine and nucleotides, were reduced in M1 macrophages. Moreover, the mRNA expression of the potential transporter (*Slc1a2*) for Asp uptake was significantly upregulated in M1 macrophages, indicating that macrophages may require extra Asp for M1 polarization.

The polarization of macrophages is regulated by multiple signaling pathways. mTOR can be activated by amino acids to promote the polarization of M1 macrophages (10). Our data showed that mTOR was activated by Asp, which might be due to the role of Asp on NEAA synthesis through transamination, which is consistent with the result that Asp increases the levels of intracellular and supernatant amino acids (4). NF-κB is a crucial transcription factor regulating the expressions of inflammatory cytokines (31). In this study, we found that Asp had little effect on the NF-κB pathway, while asparagine promoted the activation of NF-κB. This is possibly due to intracellular asparagine being an exchange factor for extracellular amino acids (e.g., serine) (24). This is consistent with our previous study, which showed that intracellular serine contributed to the activation of NF-κB (10). Other possible reasons may be the inefficient transport of Asp, as 10 mM Asp and 1mM Asn increased intracellular asparagine to 73.83% and 116.4%, respectively, indicating that the activation of NF-κB may be mainly induced by asparagine. Moreover, the NF-κB pathway and the TCA cycle intermediate succinate could regulate HIF-1α transcription to drive the production of pro-inflammatory cytokines (49). Succinate is synthesized by glutamate through α-ketoglutaric acid (α-KG) in M1 macrophages (50), and asparagine increases the intracellular glutamate in our study. However, the intracellular serine, α-KG, and succinate were not detected in this study. Whether Asp and asparagine increase the activity of HIF-1α through glutamate/α-KG/succinate metabolic pathways in M1 macrophages warrants further investigation. In addition, Asp and asparagine both increase the level of adenosine, and the adenosine A_2a_ receptor promotes the activation of HIF-1α through the PKC and PI3K pathways in macrophages (51), indicating that Asp might increase the activity of HIF-1α through nucleotide metabolism. In addition to nucleotides, amino acid metabolism is also involved in the activation of HIF-1α (e.g., glutamine) (15, 52), and we found that phenylalanine inhibited the activation of HIF-1α (unpublished), but it remains to be seen how this contributes to the regulation of Asp in HIF-1α.

As cytosolic sensors of microbial molecules, Nod-like receptors (NLRs) support host defense against microbial pathogens and regulate immune homeostasis in various tissues (53). Among the NLRs, the NLRP3 inflammasome is the best characterized to date (54). Aspartate metabolism induces the activation of NLRP3/caspase-1 inflammasome, which might promote the activation of HIF-1α (55). In addition, the activation of NLRP3 inflammasome complex is required for the synthesis and oxidation of mitochondrial DNA (mtDNA), and the translocation of nucleoside monophosphate kinase CMPK2 to the mitochondria enhances the supply of deoxyribonucleoside triphosphates (dNTPs) for mtDNA synthesis (56). Consistently, the mRNA expression of *CMPK2* was increased in the M1 macrophages in this study, which could be further enhanced by asparagine. However, this study lacks evidence to identify whether *CMPK2* is involved in the effect of Asp metabolism on directing the polarization of M1 macrophages; therefore, whether asparagine or Asp regulates these complicated cellular activities through mtDNA synthesis remains to be determined.

Aspartate is a critical precursor in nucleotide, protein, and asparagine synthesis. Nucleoside metabolites have been reported to be involved in the polarization of macrophages, such as the inhibition of inosine and adenosine in M1 macrophages (57, 58). Although M1 macrophages specifically release cytosine and deoxycytidine extracellularly in this study, we found that these nucleotides inhibit M1 macrophage polarization. It is worth mentioning that providing the alternative Asp product, asparagine, could promote M1 polarization through metabolic remodeling and NLRP3/caspase-1 activation. Additionally, Asp and asparagine promoted the biosynthesis of aminoacyl-tRNA, which is an essential biological process responsible for charging amino acids to their cognate tRNAs and providing the substrates for global protein synthesis (36). Interestingly, the translation inhibitor blocked the effects of Asp and asparagine. Therefore, Asp might promote the polarization of M1 macrophages through the regulation of translation and the synthesis of asparagine.

A previous study showed that Asp downregulates the production of IL-1β (59), which are contrary to the results of our study. However, this study induced an acute reaction of macrophages through activating the adenosine triphosphate (ATP)-gated ionotropic P2X7 receptors (P2X7R) by LPS and ATP, and extracellular Asp acted on the membrane receptors (*N*-methyl-d-aspartate receptors) to regulate acute reaction within 1 h. We induced the inflammatory macrophages with LPS and IFN-γ, and Asp promoted the production of IL-1β through intracellular metabolism in 15 h. Differences in the experimental methods and the regulatory mechanism may be the reason for the different results observed. In addition, Asp can provide oxaloacetate, α-ketoglutarate, fumarate, and glutamate by supporting NEAA synthesis through transamination or recycle NAD^+^ through the malate–Asp shuttle in order to fuel the TCA cycle (4). Notably, previous studies have reported that purine governs the switch from the pro-inflammatory to the suppressive phenotype (60), suggesting that Asp may promote M2 activation by fueling the TCA cycle and producing purine metabolites. However, a recent study has shown that the inhibition of the synthesis of Asp by aminooxyacetic acid (AOAA, an inhibitor of Asp aminotransferase) restrains pro-inflammatory M1 macrophages while it promotes the anti-inflammatory M2 phenotype (50), indicating that Asp may prejudice the polarization of M2 macrophages. Therefore, the function of Asp in M2 polarization is still controversial.

Due to the potential functions of Asp and its metabolites in a variety of immune cells, it is feasible to regulate the function of immune cells by targeting Asp metabolism in order to influence infection, cancer, and other diseases. Here, we found that Asp inhibited the colonization of *C. rodentium* in the colon. Although Asp and its metabolites play a role in promoting the pro-inflammatory polarization of macrophages and T-cell activation (20, 44), as it is also an important substrate for protein and nucleotide synthesis, numerous reports have shown that Asp leads to the proliferation of breast cancer cells (61) and other cancer cells (22). Therefore, the application of Asp in cancers is interesting. For instance, limiting the supply of endogenous Asp (47) or exogenous asparagine (23) can inhibit tumor growth; however, providing asparagine to mice also enhances the tumor-suppressive effect of OT-I CD8^+^ T cells (43). Therefore, the role of Asp in the targeted treatment of cancer may be complex, but it still has a strong application prospect, which is worth further exploration for its immune regulation function and application in the treatment of diseases.

In conclusion, our results have revealed the metabolic cues of Asp in M1 macrophages. More importantly, Asp promotes the IL-1β production of M1 macrophages through metabolic remodeling and the activation of NLRP3/caspase-1. We further confirmed that Asp enhances the bactericidal activity and intestinal immune responses *in vivo*. This study may shed light on new mechanisms of Asp metabolism in the regulation of immune responses and be helpful for its future application in immune-related diseases.

## Data Availability Statement

The original contributions presented in the study are included in the article/**Supplementary Material**. Further inquiries can be directed to the corresponding author.

## Ethics Statement

The animal study was reviewed and approved by the Administration Office of Laboratory Animals of SCAU.

## Author Contributions

WR designed the experiment. HW, XZ, BL, and YX conducted the experiment. ZX and BD analyzed the metabolites. LH and JD helped with the animal experiment. HW analyzed the data and drafted the manuscript. WR revised and approved the final manuscript. All authors contributed to the article and approved the submitted version.

## Funding

This work was supported by National Natural Science Foundation of China (31922079 and 31872365) and Guangdong Basic and Applied Basic Research Foundation (2019B1515210002).

## Conflict of Interest

The authors declare that the research was conducted in the absence of any commercial or financial relationships that could be construed as a potential conflict of interest.

## Publisher’s Note

All claims expressed in this article are solely those of the authors and do not necessarily represent those of their affiliated organizations, or those of the publisher, the editors and the reviewers. Any product that may be evaluated in this article, or claim that may be made by its manufacturer, is not guaranteed or endorsed by the publisher.

## Supplementary Material

Supplementary Figure 1 Synthesis and transportation of aspartate/asparagine. **(A)** The mRNA expressions of the enzyme for asparagine/aspartate synthesis in M0 and M1 macrophages (*n* = 3). **(B)** The mRNA expressions of the asparagine transporters in M0 and M1 macrophages (*n* = 3-4). **(C)** The mRNA expressions of the aspartate transporters in M0 and M1 macrophages (*n* = 3-4). Data analyzed with unpaired *t*-test and error bars represent mean ± SEM, **P≤* 0.05, ***P≤* 0.01.

Supplementary Figure 2 Aspartate changes the polarization and cellular metabolism of macrophages. **(A)** IL-1β and TNF-α secretion in M1 macrophages with aspartate supplementation at dosage of 0.1mM or 1mM (*n* = 4-5). **(B)** The mRNA expressions of IL-1β and TNF-α in aspartate-treated M1 macrophages (*n* = 6). **(C)** Principal component analysis (PCA) of global metabolite profiles of aspartate-treated M1 macrophages (*n* = 6). **(D)** Volcano plot of pairwise comparisons of all detected ions using an unpaired t test for the metabolome of M1 macrophages and aspartate-treated M1 macrophages (*n* = 6). **(E)** The mRNA expressions of the enzyme for nucleotide metabolism in aspartate-treated M1 macrophages (*n* = 6). **(F)** The metabolic pathways affected by aspartate in M1 macrophages (*n* = 6). Data analyzed with one-way ANOVA **(A, B)** or unpaired *t*-test **(E)** and error bars represent mean ± SEM, **P≤* 0.05, ***P≤* 0.001.

Supplementary Figure 3 Cytosine or deoxycytidine inhibits inflammatory macrophage polarization. **(A)** The fold change of supernatant cytosine, cytidine and deoxycytidine in M0 or M1 macrophages (*n* = 6). **(B)** The fold change of supernatant cytosine, cytidine and deoxycytidine in aspartate-treated M1 macrophages (*n* = 3-4). **(C, D)** The protein abundance of IL-1β, IKK, p-IKK and caspase-1 in macrophages (*n* = 4). Data analyzed with unpaired *t*-test **(A, B)** or one-way ANOVA **(C, D)** and error bars represent mean ± SEM, **P≤* 0.05.

Supplementary Figure 4 IL-1β and TNF-α secretion in asparagine-treated M1 macrophages. **(A, B)** IL-1β and TNF-α secretion in asparagine-treated M1 macrophages (*n* = 4-5). Data analyzed with one-way ANOVA and error bars represent mean ± SEM.

Supplementary Figure 5 Asparagine modulates cellular metabolism of macrophages. **(A)** Principal component analysis (PCA) of global metabolite profiles in M1 macrophages with asparagine supplementation (*n* = 6). **(B)** Volcano plot of pairwise comparisons of all detected ions using an unpaired t test for the metabolome of M1 macrophages and M1 macrophages with asparagine supplementation (*n* = 6). **(C)** The metabolic pathway affected by asparagine in macrophages (*n* = 6). **(D)** The fold change of supernatant amino acids in M1 macrophages with asparagine supplementation (*n* = 6). Data analyzed with unpaired *t*-test and error bars represent mean ± SEM. **P≤* 0.05.

Supplementary Figure 6 Effect of aspartate on macrophage responses in mice and weaning piglets. **(A)** The body weight of control mice and mice with 0.5% aspartate supplementation at day 10 of post-infection (*n* = 11). **(B)** The length of colon in control mice and mice with 0.5% aspartate supplementation (*n* = 11). **(C)** The mRNA expressions of IL-1β and TNF-α in the ileum and colon of control piglets and piglets with aspartate supplementation for 28 days (*n* = 5). **(D)** The mRNA expressions of IL-1β and TNF-α in the ileum and colon of control piglets and piglets with aspartate supplementation for 90 days (*n* = 6). Data analyzed with unpaired *t*-test **(A, B)** or one-way ANOVA **(C, D)** and error bars represent mean ± SEM.

Supplementary Table 1 Primers used for mRNA expression analysis by RT-PCR.
