# Supplementary figures and images for: Aspartate Metabolism Facilitates IL-1β Production in Inflammatory Macrophages
Source: Front Immunol. 2021 Oct 21;12:753092. doi: 10.3389/fimmu.2021.753092 (PMC8567039; doi:10.3389/fimmu.2021.753092)

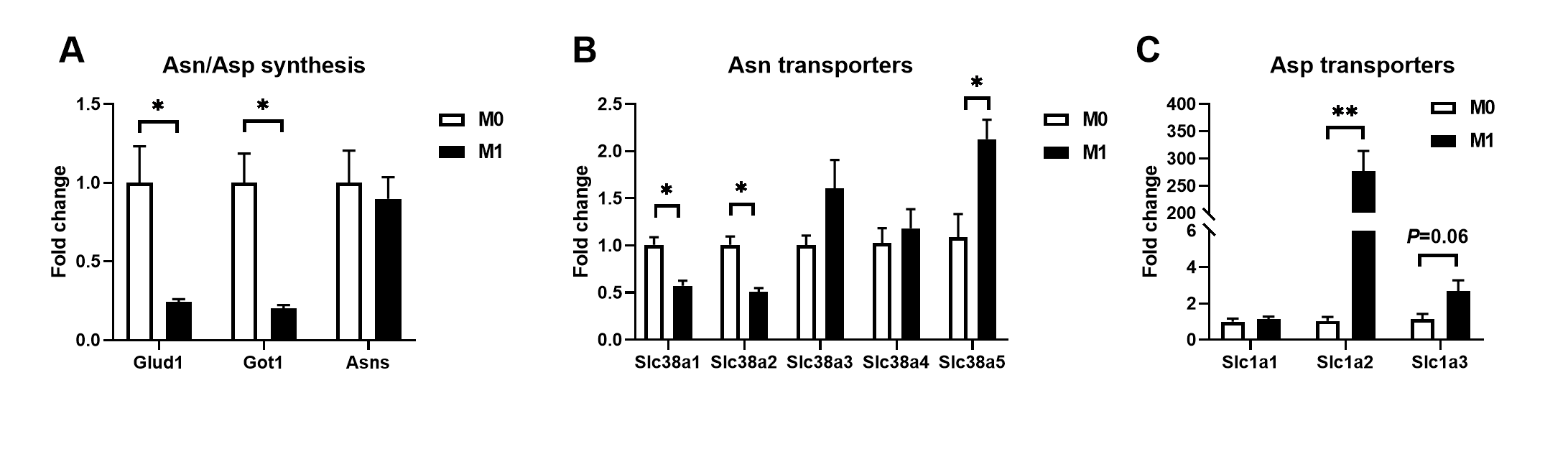

Supplement: Supplementary Figure 1 — Synthesis and transportation of aspartate/asparagine. (A) The mRNA expressions of the enzyme for asparagine/aspartate synthesis in M0 and M1 macrophages (n = 3). (B) The mRNA expressions of the asparagine transporters in M0 and M1 macrophages (n = 3-4). (C) The mRNA expressions of the aspartate transporters in M0 and M1 macrophages (n = 3-4). Data analyzed with unpaired t-test and error bars represent mean ± SEM, *P≤ 0.05, **P≤ 0.01. [file Image_1.tif]

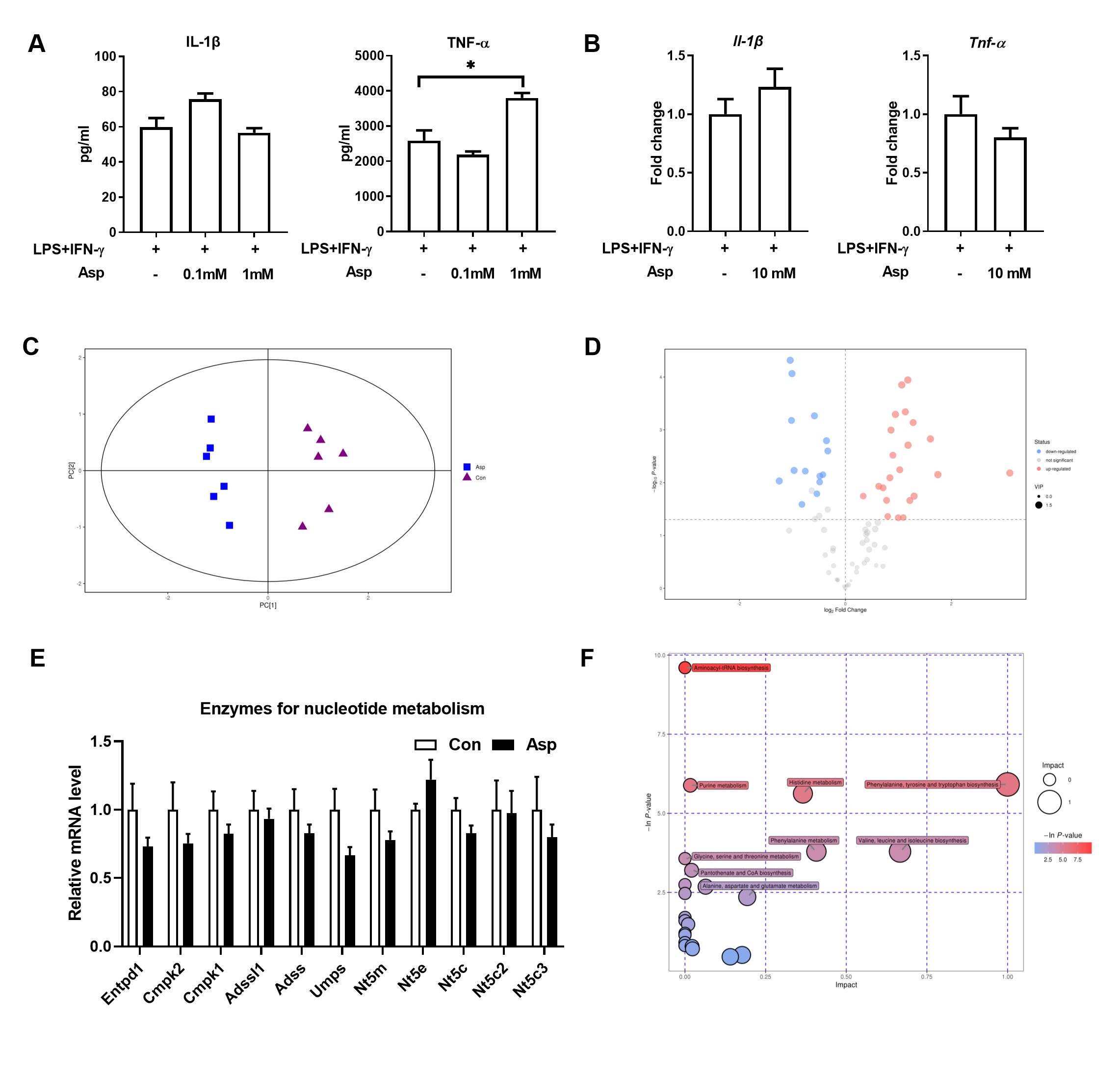

Supplement: Supplementary Figure 2 — Aspartate changes the polarization and cellular metabolism of macrophages. (A) IL-1β and TNF-α secretion in M1 macrophages with aspartate supplementation at dosage of 0.1mM or 1mM (n = 4-5). (B) The mRNA expressions of IL-1β and TNF-α in aspartate-treated M1 macrophages (n = 6). (C) Principal component analysis (PCA) of global metabolite profiles of aspartate-treated M1 macrophages (n = 6). (D) Volcano plot of pairwise comparisons of all detected ions using an unpaired t test for the metabolome of M1 macrophages and aspartate-treated M1 macrophages (n = 6). (E) The mRNA expressions of the enzyme for nucleotide metabolism in aspartate-treated M1 macrophages (n = 6). (F) The metabolic pathways affected by aspartate in M1 macrophages (n = 6). Data analyzed with one-way ANOVA (A, B) or unpaired t-test (E) and error bars represent mean ± SEM, *P≤ 0.05, **P≤ 0.001. [file Image_2.tif]

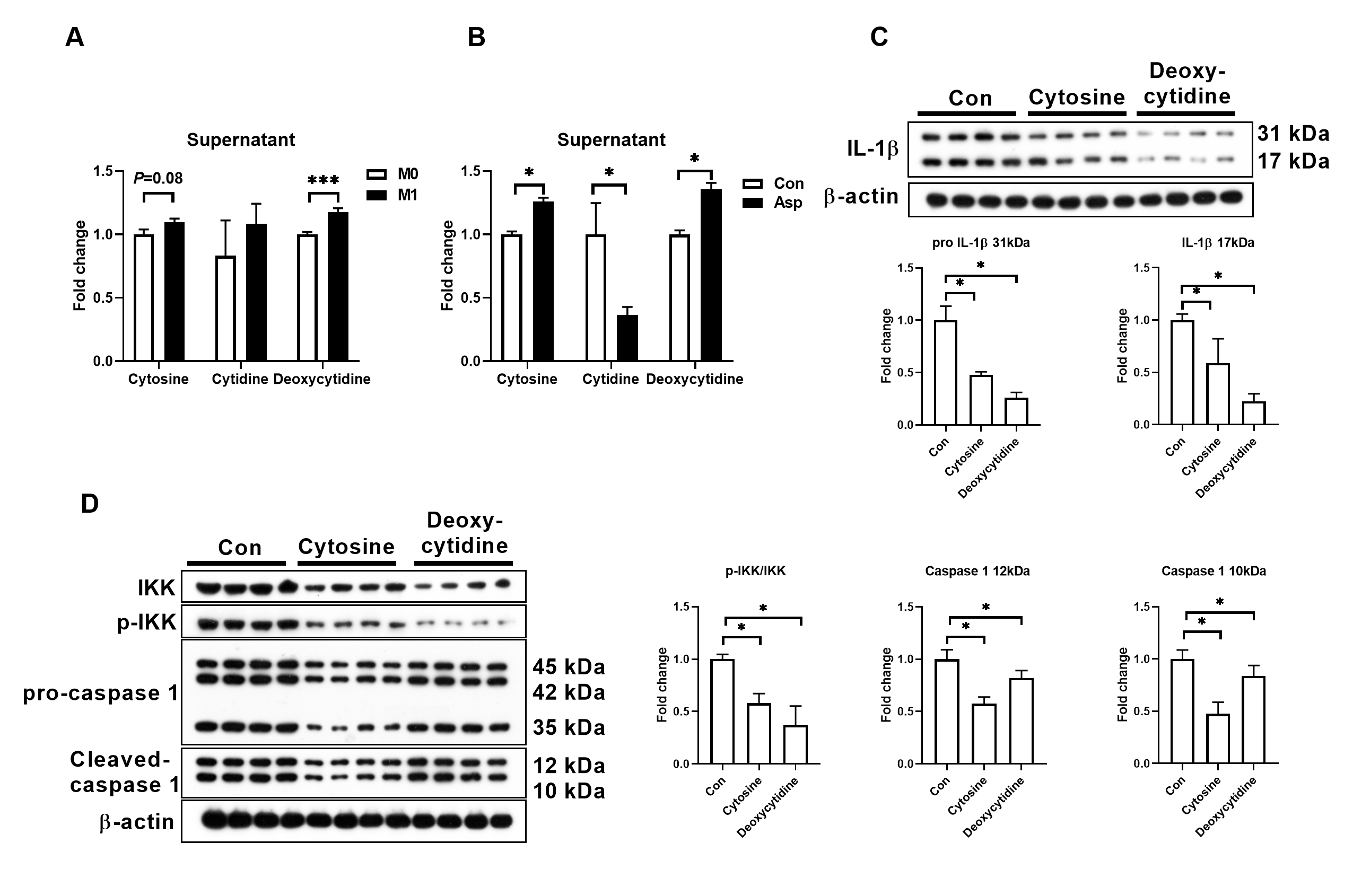

Supplement: Supplementary Figure 3 — Cytosine or deoxycytidine inhibits inflammatory macrophage polarization. (A) The fold change of supernatant cytosine, cytidine and deoxycytidine in M0 or M1 macrophages (n = 6). (B) The fold change of supernatant cytosine, cytidine and deoxycytidine in aspartate-treated M1 macrophages (n = 3-4). (C, D) The protein abundance of IL-1β, IKK, p-IKK and caspase-1 in macrophages (n = 4). Data analyzed with unpaired t-test (A, B) or one-way ANOVA (C, D) and error bars represent mean ± SEM, *P≤ 0.05. [file Image_3.tif]

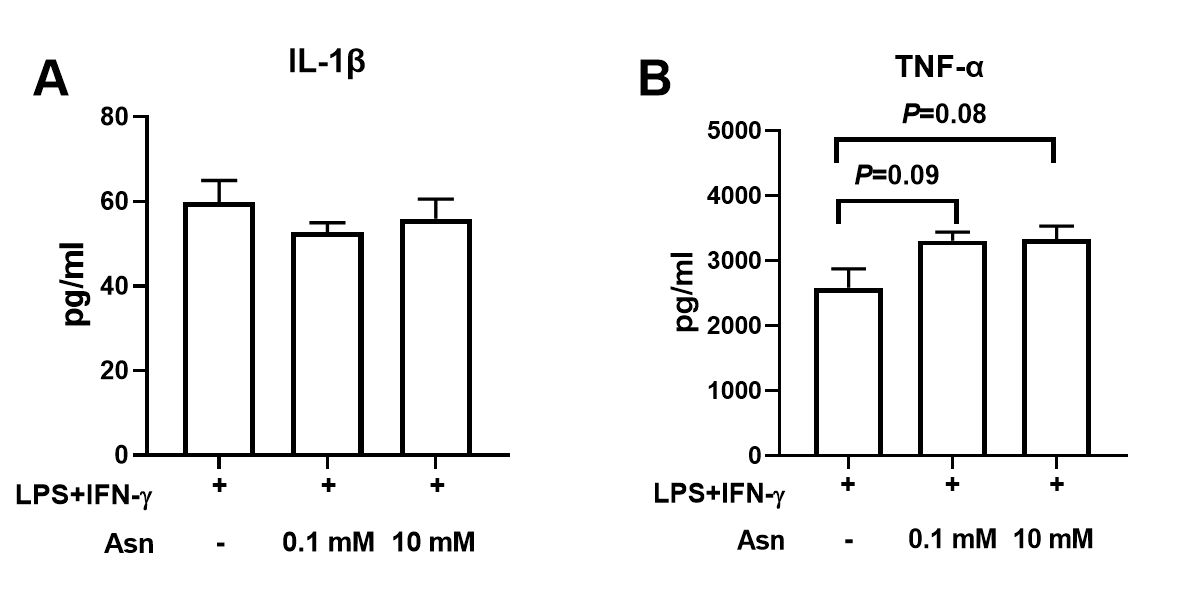

Supplement: Supplementary Figure 4 — IL-1β and TNF-α secretion in asparagine-treated M1 macrophages. (A, B) IL-1β and TNF-α secretion in asparagine-treated M1 macrophages (n = 4-5). Data analyzed with one-way ANOVA and error bars represent mean ± SEM. [file Image_4.tif]

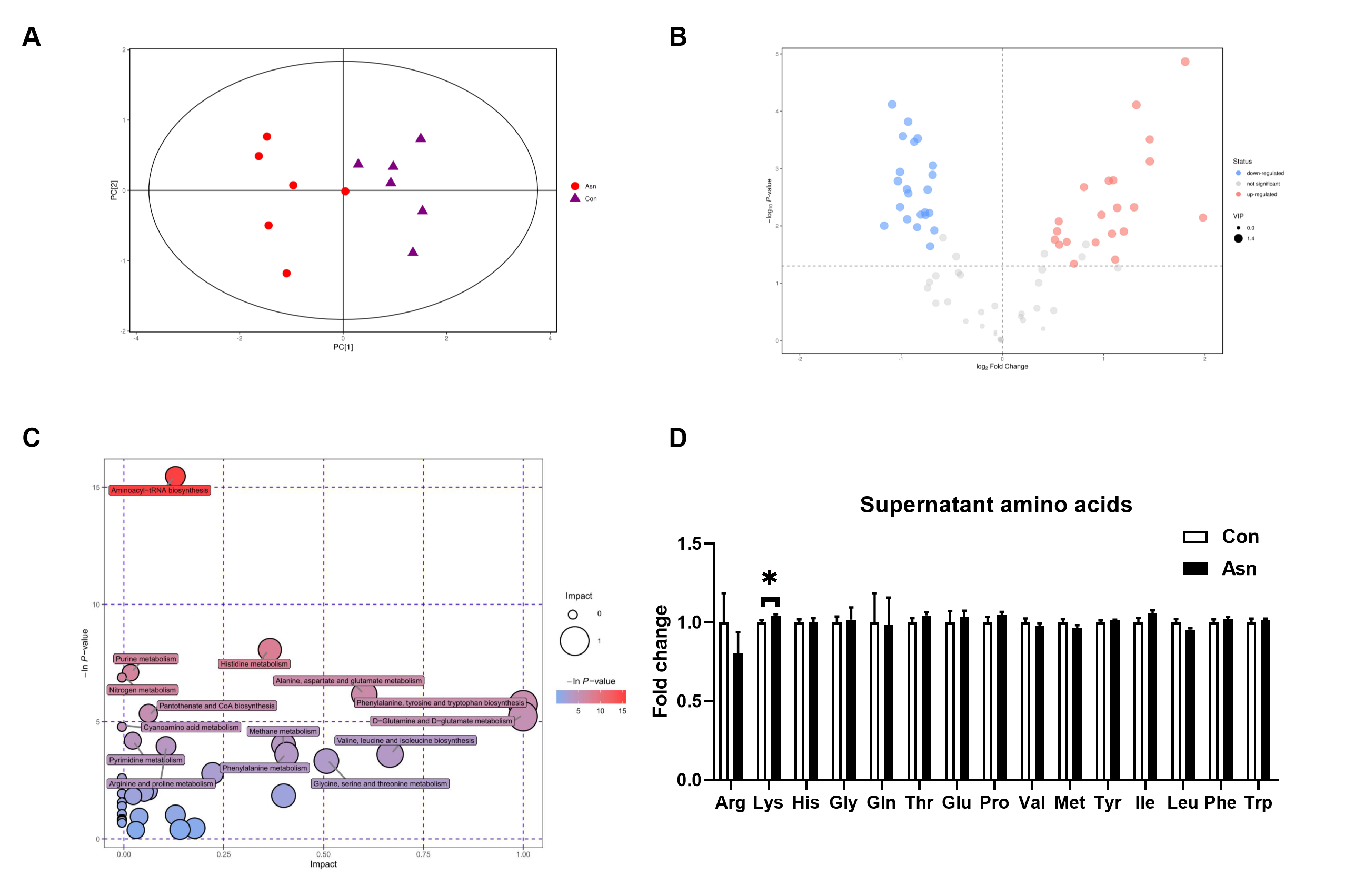

Supplement: Supplementary Figure 5 — Asparagine modulates cellular metabolism of macrophages. (A) Principal component analysis (PCA) of global metabolite profiles in M1 macrophages with asparagine supplementation (n = 6). (B) Volcano plot of pairwise comparisons of all detected ions using an unpaired t test for the metabolome of M1 macrophages and M1 macrophages with asparagine supplementation (n = 6). (C) The metabolic pathway affected by asparagine in macrophages (n = 6). (D) The fold change of supernatant amino acids in M1 macrophages with asparagine supplementation (n = 6). Data analyzed with unpaired t-test and error bars represent mean ± SEM. *P≤ 0.05. [file Image_5.tif]

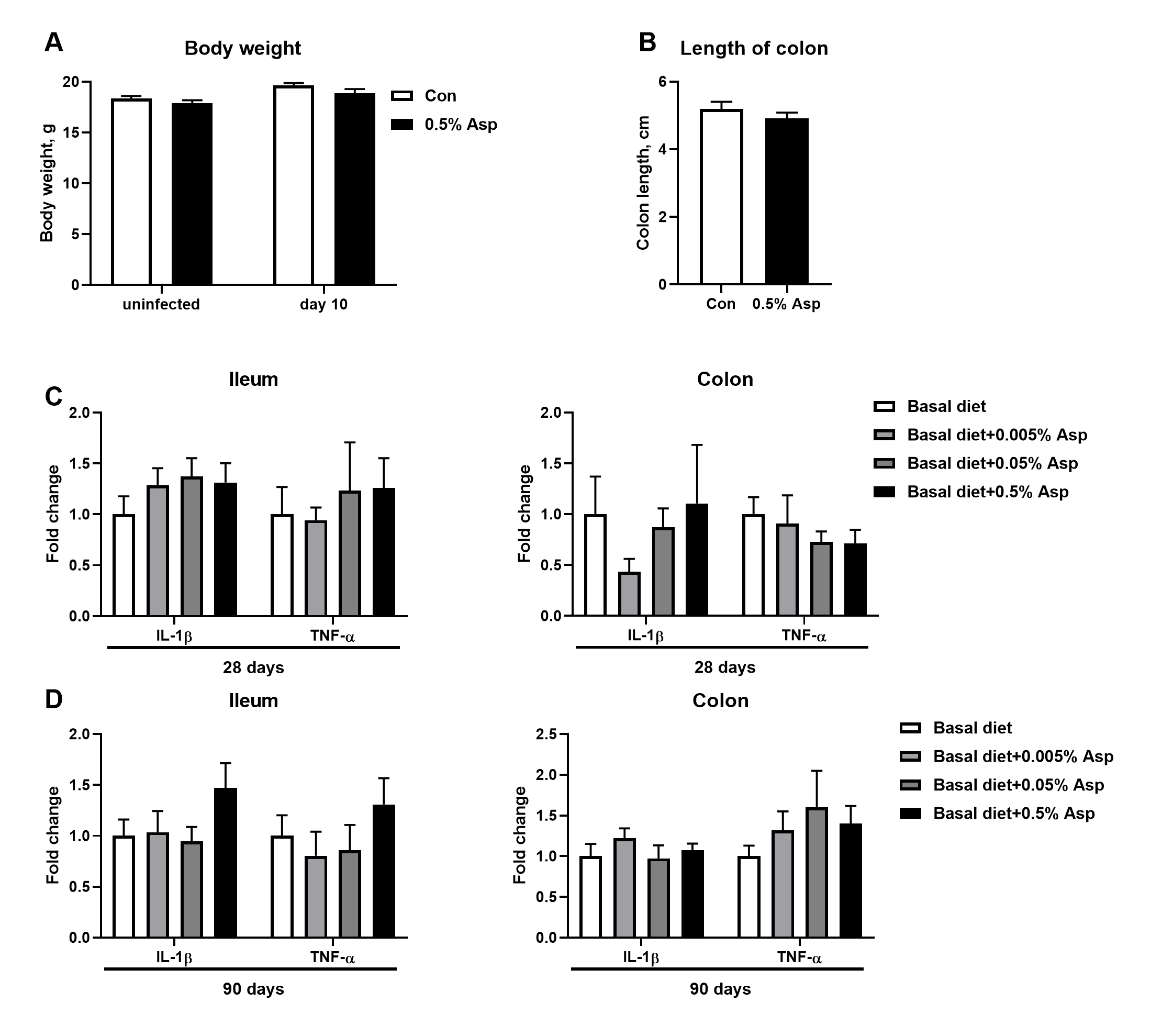

Supplement: Supplementary Figure 6 — Effect of aspartate on macrophage responses in mice and weaning piglets. (A) The body weight of control mice and mice with 0.5% aspartate supplementation at day 10 of post-infection (n = 11). (B) The length of colon in control mice and mice with 0.5% aspartate supplementation (n = 11). (C) The mRNA expressions of IL-1β and TNF-α in the ileum and colon of control piglets and piglets with aspartate supplementation for 28 days (n = 5). (D) The mRNA expressions of IL-1β and TNF-α in the ileum and colon of control piglets and piglets with aspartate supplementation for 90 days (n = 6). Data analyzed with unpaired t-test (A, B) or one-way ANOVA (C, D) and error bars represent mean ± SEM. [file Image_6.tif]
