# Supplementary material for: Aspartate Metabolism Facilitates IL-1β Production in Inflammatory Macrophages
Source: Front Immunol. 2021 Oct 21;12:753092. doi: 10.3389/fimmu.2021.753092 (PMC8567039; doi:10.3389/fimmu.2021.753092)
Supplement: Supplementary Table 1 — Primers used for mRNA expression analysis by RT-PCR. [file Table_1.docx]

**TABLE S1 |** Primers used for mRNA expression analysis by RT-PCR.

| **Gene** | **Forward Primer (5’ to 3’)** | **Reverse Primer (5’ to 3’)** |
| --- | --- | --- |
| β-actin | GTCCCTCACCCTCCCAAAAG | GCTGCCTCAACACCTCAACCC |
| IL-1β | GCAACTGTTCCTGAACTCAACT | ATCTTTTGGGGTCCGTCAACT |
| TNF-α | GCAGCCTTGTCCCTTGAAGA | ACGTCGTAGCAAACCACCAA |
| Asns | TTACCTGTCTCTGCCGCCAGAT | CACTGAAGGCTTCTTTGGGTCG |
| Adsl | CCGACTTTGCTAAGGATCGTGC | CACGCTTCAAGTTCTGGAGATCC |
| Adss | GGTATCCAGGAACAGCAGAGAC | TCACACATCCGAAGTCCACTCC |
| Asll1 | GATGATCCTCCGATACGCTCAC | CCGTTGAGCTTGTAGGAGATGC |
| Umps | TGGCGACAGTTATCTGCTCAGC | CGTCCTCAATGACCAGACAGGT |
| Cmpk1 | TGAAGCCGTTGGTCGTGTTCGT | GAAGCTCTCCTGCAGAAAGGTG |
| Cmpk2 | AACTCTGCGGTGTTCCAAGACC | GGAACTTCCCTTTCTGGACCTC |
| Ctps2 | CAAAGCACTGGAACACTCAGCC | GCTAAGCACAGCTTCTGCCAAG |
| Gmps | CCTTGTTGCCAGTGGTAAAGCC | TCTTCTGGCAGGTCAAGCTCTC |
| Impdh1 | CTGTGGTTCCATCTGCATCACC | GATGCCACCATCCGCTATTACC |
| Impdh2 | CTTGCTGGTGTGGATGTAGTGG | GCCTCCAATGACCTGTAGACTG |
| Glud1 | TCCGTTACAGCACTGACGTGAG | ACGCCTGCTTTAGCACCTCCAA |
| Got1 | GCGCCTCCATCAGTCTTTG | ATTCATCTGTGCGGTACGCTC |
| Slc38a1 | GCAGAACTCGACAGTCAGTGCTA | GCGATGGTTGGTAAAGCATACA |
| Slc38a2 | TGCAGGCCACGCTATTTCA | GGCTGGCGGCTCTTTAGCTCT |
| Slc38a3 | GCTGCCCATATATACAGAGCTCAA | CAGCAATGGACAGGTTGGAGAT |
| Slc38a4 | AGCTACACCGGCATGGAAAA | CTCATCCGCGTAATCGGCTA |
| Slc38a5 | TGGAGGTGTCTGGTCTCTAATAA | GGCAGTGAGGCAACTCTAAGG |
| Slc1a1 | AGCATGATCACAGGTGTCG | CTCACAACTAACACAATACCTAGG |
| Slc1a2 | TGCTCATCCTCCCTCTTATCATC | GGCCGCTGGCTTTAGCAT |
| Slc1a3 | TGGCAGTCATTTTTCGACAC | ATGGACCAGCAAAAGGATCA |
| Entpd1 | CTGGACAAGAGGAAGGTGCCTA | GACTGTCTGAGATGAGGCTTAGC |
| Adssl1 | GATGATCCTCCGATACGCTCAC | CCGTTGAGCTTGTAGGAGATGC |
| Nt5m | CCGTTGAGCTTGTAGGAGATGC | GCAGGAGGTAAAGAGGATGTGC |
| Nt5e | CGCTCAGAAAGTTCGAGGTGTG | CGCAGGCACTTCTTTGGAAGGT |
| Nt5c | GCCAGAGTTTGTGGAGCGGATT | GCAGGTGAACAAGATGTGCTCC |
| Nt5c2 | GTGGCAGTCCTACTTTGACCTG | CCTGAGTAGACAATGCCGTGCT |
| Nt5c3 | GTTGATCCTGTTCTCACCGTGG | CGTCAGAGTCAGCCACAATCTC |
